# Protective Effects of 5-MTP in a Rat Model of Diabetic Cardiomyopathy Through Anti-Inflammatory, Anti-Apoptotic, and Antifibrotic Mechanisms

**DOI:** 10.3390/life16071198

**Published:** 2026-07-20

**Authors:** Susetyo Atmojo, Bambang Budi Siswanto, Nurjati Chairani Siregar, Aria Kekalih, Fadlina Chany Saputri, Budi Susetyo Pikir, Deni Noviana, Apridya Nurhafizah, Wilbert Huang, Puspita Eka Wuyung

**Affiliations:** 1 Doctoral Program in Medical Sciences, Faculty of Medicine, Universitas Indonesia, Jakarta 16424, Indonesia; 2 National Cardiovascular Center Harapan Kita, Jakarta 11420, Indonesia; napridya@gmail.com (A.N.); wilberthuang67@gmail.com (W.H.); 3 Department of Cardiology and Vascular Medicine, Faculty of Medicine, Universitas Indonesia, Jakarta 16424, Indonesia; 4 Department of Anatomical Pathology, Faculty of Medicine, Universitas Indonesia, Jakarta 10430, Indonesia; 5 Dr. Cipto Mangunkusumo General Hospital, Jakarta 16424, Indonesia; 6 Department of Community Medicine, Faculty of Medicine, Universitas Indonesia, Jakarta 16424, Indonesia; 7 Department of Pharmacology-Toxicology, Faculty of Pharmacy, Universitas Indonesia, Jakarta 16424, Indonesia; 8 Department of Cardiology and Vascular Medicine, Faculty of Medicine, Universitas Airlangga, Surabaya 60132, Indonesia; 9 Dr. Soetomo General Academic Hospital–Universitas Airlangga Hospital, Surabaya 60132, Indonesia; 10 Division of Surgery and Radiology, School of Veterinary Medicine and Biomedical Sciences, IPB University, Bogor 16680, Indonesia; 11 Animal Research Facility, Indonesia Medical Education and Research Institute (IMERI), Faculty of Medicine, Universitas Indonesia, Jakarta 10430, Indonesia

**Keywords:** diabetic cardiomyopathy, 5-methoxytryptophan, myocardial remodeling

## Abstract

Background: Diabetic cardiomyopathy (DCM) is characterized by myocardial inflammation, apoptosis, and fibrosis that contribute to ventricular remodeling and dysfunction. We investigated the effects of 5-methoxytryptophan (5-MTP) on histopathological and molecular markers of myocardial remodeling in a rat model of DCM. Methods: Forty-eight Sprague–Dawley rats with DCM induced by a high-fat high-fructose diet and low-dose streptozotocin (25 mg/kg) were randomized to control or 5-MTP treatment (25, 50, or 100 mg/kg) and evaluated after 8, 16, and 32 days. Histopathological assessment using hematoxylin–eosin and Masson’s trichrome staining, along with immunohistochemical analysis of inflammatory, apoptotic, and fibrotic markers, was performed. Results: Myocardial inflammatory histopathological scores did not differ significantly among groups. Myocardial fibrosis assessed by Masson’s trichrome staining was significantly reduced at day 16 (*p* = 0.020), with all 5-MTP doses demonstrating lower fibrosis scores than DCM controls. Collagen I expression did not differ significantly. Caspase-3 expression was significantly reduced in all treatment groups at day 16 (*p* = 0.029), with persistent reduction at day 32 only in the 100 mg/kg group (*p* = 0.004). Early molecular modulation was observed through reduced TGF-β expression at day 8 in the 25 mg/kg and 50 mg/kg groups, followed by reduced SMAD3 expression at day 16 in the 25 mg/kg and 100 mg/kg groups. At day 16, AKT expression increased in the 25 mg/kg group, while cytoplasmic NF-κB expression decreased in the 25 mg/kg and 100 mg/kg groups. Conclusion: 5-MTP demonstrated time-dependent changes in molecular and histopathological markers associated with myocardial remodeling in experimental DCM.

## 1. Introduction

Diabetes mellitus (DM) remains a major global health burden and is strongly associated with cardiovascular morbidity and mortality. Epidemiological studies suggest that the growing incidence of DM is driven by the increase of obesity, westernized lifestyle, and global economic growth. Beyond atherosclerotic complications, diabetes directly affects myocardial structure and function, contributing to the development of diabetic cardiomyopathy, a distinct myocardial disorder characterized by ventricular remodeling and systolic and diastolic dysfunction independent of coronary artery disease, hypertension, or valvular disease [1,2].

The pathogenesis of DCM is complex and involves chronic inflammation, cardiomyocyte apoptosis, and myocardial fibrosis. Hyperglycemia-induced metabolic stress promotes activation of proinflammatory signaling pathways, including nuclear factor kappa B (NF-κB), leading to increased cytokine production and myocardial injury. In parallel, activation of apoptotic pathways such as caspase-3 contributes to cardiomyocyte loss, while profibrotic signaling, mediated by transforming growth factor-β (TGF-β) and its downstream pathways, drives extracellular matrix deposition and ventricular remodeling [3,4,5].

An endogenous metabolite of tryptophan, 5-Methoxytryptophan shows emerging anti-inflammatory and antifibrotic properties. Previous studies have demonstrated that 5-MTP inhibits macrophage activation, suppresses TGF-β signaling, and reduces collagen deposition in various disease models [6,7]. However, its role in modulating the molecular mechanisms underlying DCM remains unclear.

Therefore, this study aimed to investigate the effects of 5-MTP on histopathological and molecular markers related to myocardial inflammation, apoptosis, and fibrosis in an experimental model of diabetic cardiomyopathy with a focus on key signaling pathways involved in disease progression.

## 2. Materials and Methods

### 2.1. Study Design and Sample Size

**Figure 1:**
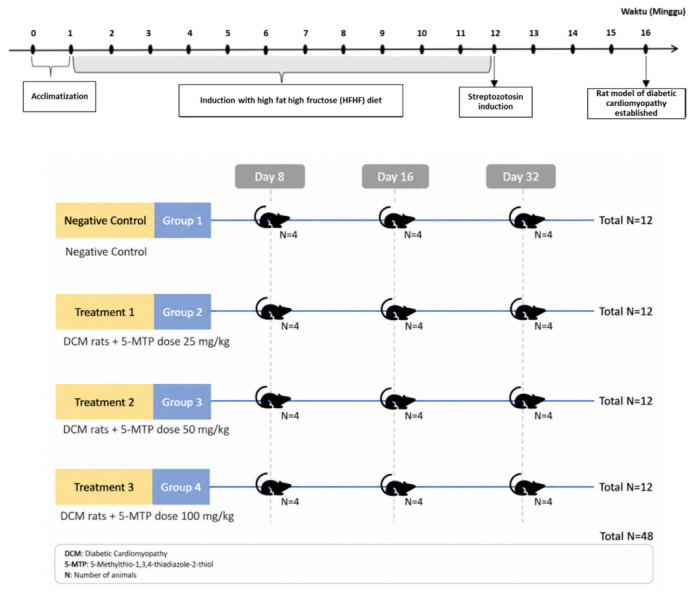
Study design of effects of different dose 5-MTP on rat model of diabetic cardiomyopathy on different treatment duration.

This was an in vivo experimental study using a randomized parallel-group design to evaluate the effects of 5-methoxytryptophan in a rat model of diabetic cardiomyopathy. Simple random sampling methods were used to assign the rats into the groups. Rat models of DCM were randomly allocated into four groups: a DCM + vehicle control and three intervention groups receiving 5-MTP at doses of 25 mg/kg, 50 mg/kg, and 100 mg/kg. Each treatment group was further stratified according to intervention duration into 8-day, 16-day, and 32-day follow-up subgroups. Each main group consisted of 12 rats, with four rats assigned to each time-point subgroup, yielding a total of 48 rats across all groups. Baseline evaluation, including body weight, body length, and fasting blood glucose measurement, was performed at study initiation, followed by subsequent histopathological and immunohistochemical assessments at each designated follow-up time point (Figure 1).

### 2.2. Establishment of Diabetic Cardiomyopathy Rat Model

Male Rattus norvegicus Sprague–Dawley rats aged 7–8 weeks, with an initial body weight of approximately 200–250 g, were used in this study. Prior to DCM induction, rats underwent a 7-day acclimatization period during which they were fed a standard chow diet. A rat model of diabetic cardiomyopathy was established through administration of a high-fat high-fructose (HFHF) diet ad libitum for 12 weeks, followed by a single intraperitoneal injection of streptozotocin (STZ) at a dose of 25 mg/kg, adapted from previously published HFHF/HFD-STZ models of diabetic cardiomyopathy [8]. Successful induction of diabetes was confirmed when fasting blood glucose levels exceeded 200 mg/dL at 3 days after STZ injection. Prior to initiation of the present intervention study, the HFHF/STZ protocol had been characterized in a separate pilot model-characterization study using the same dietary and streptozotocin induction protocol. The corresponding model-characterization data are provided in Supplementary S1, Tables S1 and S2, Figure S1. After completion of the designated follow-up period, rats were euthanized with ketamine at a dose of 15 mg/kg.

### 2.3. 5-Methoxytryptophan

5-MTP (Sigma-Aldrich, St. Louis, MO, USA) treatment was initiated at week 12 following successful establishment of the DCM rat model. Rats assigned to the treatment groups received intraperitoneal 5-MTP at doses of 25 mg/kg, 50 mg/kg, or 100 mg/kg every two days for predetermined treatment durations of 8, 16, or 32 days. Rats in the DCM + vehicle control group received phosphate-buffered saline via intraperitoneal injection at the same volume and administration frequency as the 5-MTP-treated groups. Before administration, 5-MTP was weighed according to the assigned dose and individual body weight of each rat, then dissolved in sterile phosphate-buffered saline (PBS; pH 7.4) prior to injection.

### 2.4. Histopathological Examination

Myocardial tissues were harvested from rat hearts, rinsed with distilled water, and fixed in 10% neutral buffered formalin. The samples were subsequently embedded in paraffin and sectioned at a thickness of 5 μm. The sections were stained with hematoxylin and eosin (HE) and Masson’s trichrome (MT), then examined under a light microscope.

H&E-stained sections were evaluated semi-quantitatively for myocardial structural alterations, including cardiomyocyte degeneration, cardiomyocyte hypertrophy, myocardial disarray, fatty infiltration, and inflammatory cell infiltration. Each parameter was graded using a four-point scale: 0 (normal), 1 (mild), 2 (moderate), and 3 (severe). A total histopathological score was calculated by summing all parameter scores, representing the overall degree of myocardial injury.

MT-stained sections were analyzed to assess myocardial fibrosis and collagen deposition. Five sections from each heart were evaluated, and the mean percentage of fibrotic involvement across all sections was calculated.

### 2.5. Immunohistochemical Studies

Immunohistochemical staining was performed to evaluate the expression of inflammatory, apoptotic, and fibrotic markers in myocardial tissue, including nuclear factor kappa B (NF-κB), caspase-3, transforming growth factor-β (TGF-β), SMAD3, protein kinase B (AKT), and collagen I.

Paraffin-embedded myocardial tissue sections (3 μm thickness) were deparaffinized in xylene and rehydrated through graded ethanol solutions. Antigen retrieval was performed using Tris–EDTA buffer (pH 9) at 95 °C followed by cooling to room temperature and washing in phosphate-buffered saline (PBS). Endogenous peroxidase activity and nonspecific protein binding were blocked prior to antibody incubation.

Sections were incubated with primary antibodies against NF-κB p65 (1:200; Cell Signaling Technology, Danvers, MA, USA), caspase-3 (1:300; Cell Signaling Technology, Danvers, MA, USA), TGF-β (1:300; Invitrogen, Carlsbad, CA, USA), AKT (1:250; Abcam, Cambridge, UK), SMAD3 (1:300; Invitrogen, Carlsbad, CA, USA), and collagen I (1:100; Invitrogen, Carlsbad, CA, USA). After incubation, the sections were treated with post-primary reagents and polymer detection system, followed by visualization using diaminobenzidine (DAB) chromogen. The slides were counterstained with hematoxylin, dehydrated, cleared, and mounted.

The stained sections were scanned using a slide scanner (Leica Aperio GT 450, Leica Biosystems, Deer Park, IL, USA) and analyzed under ×400 magnification. The expression of NF-κB was evaluated in both nuclear and cytoplasmic compartments, caspase-3 and TGF-β in the cytoplasm, AKT in nuclear and cytoplasmic regions, SMAD3 in the cytoplasm, and collagen I in the extracellular matrix, in accordance with antibody datasheets.

### 2.6. Quantification of Immunohistochemical Expression

Protein expression levels were quantified using the H-score method with ImageJ software (version 1.54r). The H-score was calculated using the following formula:

H-score = (% weakly stained cells × 1) + (% moderately stained cells × 2) + (% strongly stained cells × 3)

where staining intensity was categorized as weak (1+), moderate (2+), or strong (3+). The average H-score was calculated for each sample based on multiple high-power fields.

### 2.7. Statistical Analysis

Baseline characteristics were presented as mean ± standard deviation (SD) for normally distributed variables and median (minimum–maximum) for non-normally distributed variables, as appropriate. Normality was evaluated using the Shapiro–Wilk test, with *p* > 0.05 considered indicative of normal distribution. Group comparisons were performed using one-way ANOVA for normally distributed variables and the Kruskal–Wallis test for non-normally distributed variables, with statistical significance defined as *p* < 0.05.

For outcomes demonstrating significant treatment effects, multivariate analysis of variance (MANOVA) was subsequently conducted to evaluate the simultaneous impact of treatment on multiple correlated parameters. Overall multivariate group differences were assessed using Wilks’ Lambda, and when significant, follow-up univariate analyses (Tests of Between-Subjects Effects) were performed to determine which individual outcomes contributed to the observed differences, with Bonferroni-adjusted pairwise comparisons applied when appropriate.

## 3. Results

### 3.1. Characteristics of Rat Model of Diabetic Cardiomyopathy

A total of 48 rat models of DCM were evaluated. Mean body weight increased from 226.0 ± 18.87 g at baseline to 257.1 ± 48.97 g after high-fat high-fructose diet exposure and further to 260.0 ± 49.91 g following streptozotocin induction. During the post-intervention period, mean body weight showed a slight decrease in the early phase (251.4 ± 46.15 g at day 8 and 246.8 ± 38.42 g at day 16), followed by a modest increase to 255.9 ± 43.97 g at day 32.

Mean body length increased from 19.49 ± 1.21 cm at baseline to 20.51 ± 1.22 cm after HFHF diet exposure and reached 20.88 ± 0.91 cm following STZ induction. During the observation period after 5-MTP administration, body length remained relatively stable.

Fasting blood glucose levels increased from 95.73 ± 14.92 mg/dL at baseline to 109.7 ± 15.22 mg/dL after HFHF diet exposure, then markedly rose to 243.5 ± 88.74 mg/dL following STZ induction, confirming the establishment of the diabetic state. After 5-MTP administration, fasting glucose levels showed a gradual decline with values remained above baseline levels.

Baseline characteristics were generally comparable across treatment groups, with no significant between-group differences, except for post-STZ body weight in the 32-day cohort. Detailed baseline characteristics are provided in Supplementary Tables S3–S5.

### 3.2. Effect of 5-MTP on Myocardial Histopathological Changes

**Figure 2:**
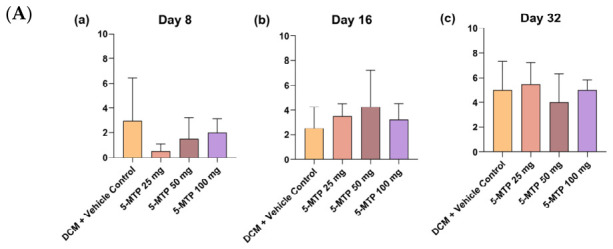

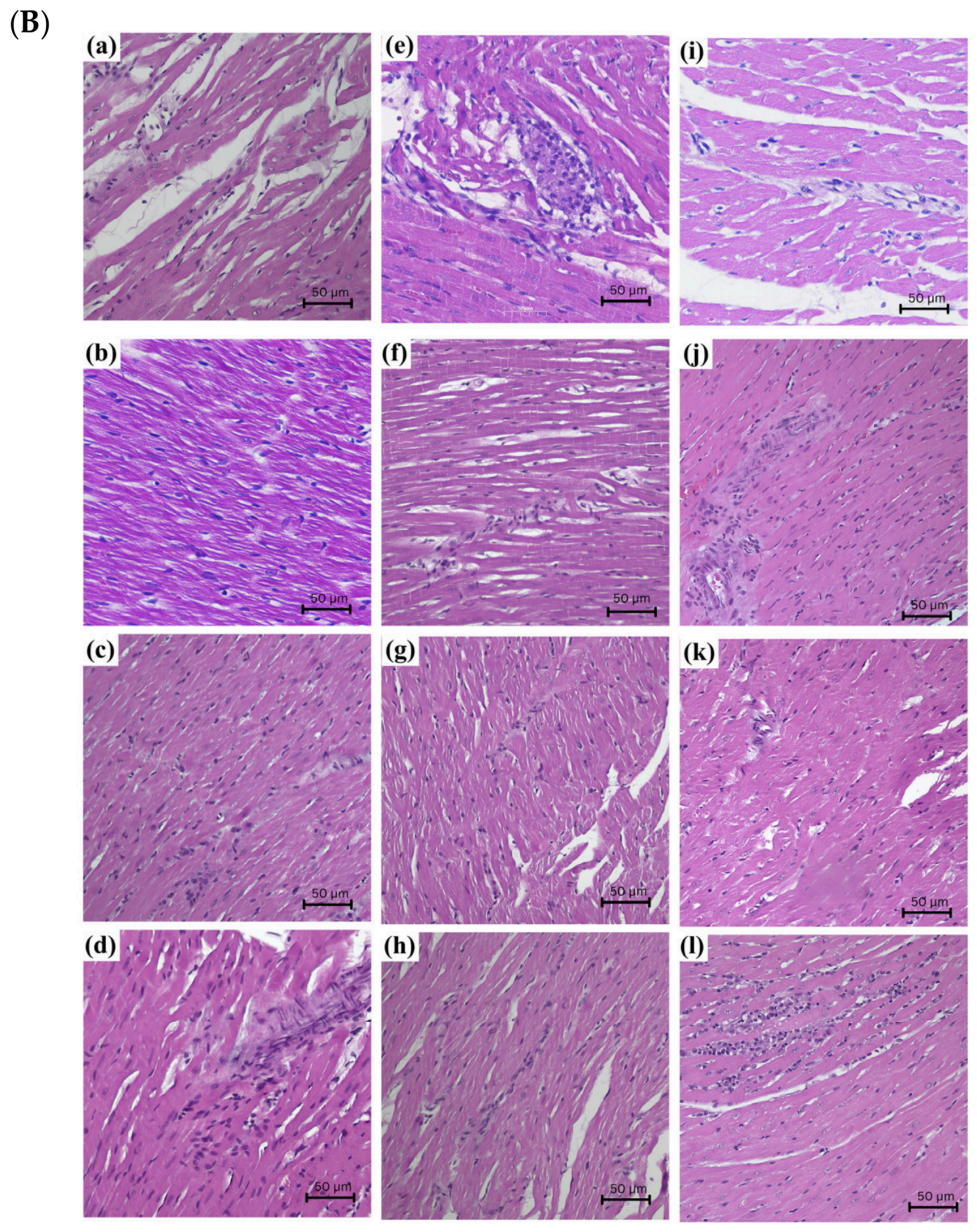
Representative hematoxylin–eosin staining and myocardial inflammatory score across treatment groups at different treatment durations. (**A**) Quantitative comparison of myocardial inflammatory scores among treatment groups at day 8 (**a**), day 16 (**b**), and day 32 (**c**). Data are presented as median (minimum–maximum) or mean ± standard deviation, as appropriate. (**B**) Representative HE-stained myocardial sections (400× magnification; scale bar = 50 μm) from each treatment group across time points: day 8 DCM + vehicle control (**a**), 5-MTP 25 mg/kg (**b**), 5-MTP 50 mg/kg (**c**), and 5-MTP 100 mg/kg (**d**); day 16 DCM + vehicle control (**e**), 5-MTP 25 mg/kg (**f**), 5-MTP 50 mg/kg (**g**), and 5-MTP 100 mg/kg (**h**); day 32 DCM + vehicle control (**i**), 5-MTP 25 mg/kg (**j**), 5-MTP 50 mg/kg (**k**), and 5-MTP 100 mg/kg (**l**). Histopathological evaluation demonstrated myocardial inflammatory cell infiltration of varying degrees without statistically significant differences among treatment groups at any time point.

In the 8-day treatment duration group, no significant difference in myocardial inflammatory score was observed among groups following 5-MTP treatment (*p* = 0.366). Similarly, no significant differences in inflammatory scores were observed at day 16 and day 32 (*p* = 0.642 and *p* = 0.727, respectively) (Figure 2).

### 3.3. Effect of 5-MTP on Myocardial Fibrosis

**Figure 3:**
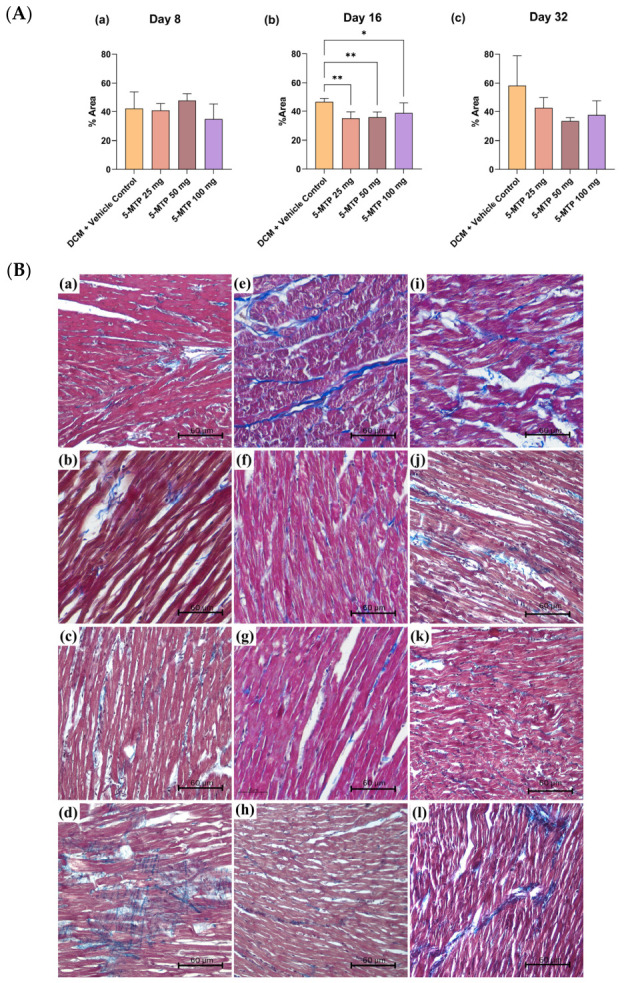
Representative Masson’s trichrome (MT) staining and quantitative myocardial fibrosis area across treatment groups at different treatment durations. (**A**) Quantitative comparison of myocardial fibrosis (% area) among treatment groups at day 8 (**a**), day 16 (**b**), and day 32 (**c**). At day 16, myocardial fibrosis differed significantly among groups, with all 5-MTP treatment groups demonstrating significantly lower fibrosis area compared with the DCM + vehicle control group (25 mg/kg, *p* = 0.005; 50 mg/kg, *p* = 0.008; 100 mg/kg, *p* = 0.037). No significant differences were observed at day 8 or day 32. * *p* < 0.05 and ** *p* < 0.01 vs DCM + vehicle control. (**B**) Representative MT-stained myocardial sections (400× magnification; scale bar = 60 μm) from each treatment group across time points: day 8 DCM + vehicle control (**a**), 5-MTP 25 mg/kg (**b**), 5-MTP 50 mg/kg (**c**), and 5-MTP 100 mg/kg (**d**); day 16 DCM + vehicle control (**e**), 5-MTP 25 mg/kg (**f**), 5-MTP 50 mg/kg (**g**), and 5-MTP 100 mg/kg (**h**); day 32 DCM + vehicle control (**i**), 5-MTP 25 mg/kg (**j**), 5-MTP 50 mg/kg (**k**), and 5-MTP 100 mg/kg (**l**). Blue staining represents collagen deposition and myocardial fibrosis. Histopathological evaluation demonstrated reduced collagen deposition following 5-MTP treatment at the intermediate time point, consistent with its transient antifibrotic effect during active myocardial remodeling.

In the 8-day treatment duration group, no significant difference in myocardial fibrosis score assessed by Masson’s trichrome staining was observed among groups (*p* = 0.246). However, in the 16-day treatment duration group, a significant difference in fibrosis score was observed among groups (*p* = 0.020), with all 5-MTP treatment groups demonstrating significantly lower fibrosis scores compared with the DCM + vehicle control group (46.6 ± 2.7), including the 25 mg/kg group (35.3 ± 4.3, *p* = 0.005), 50 mg/kg group (36.1 ± 3.5, *p* = 0.008), and 100 mg/kg group (38.8 ± 7.1, *p* = 0.037). Additionally, in the 32-day treatment duration group, no significant difference in fibrosis score was observed among groups (*p* = 0.367) (Figure 3).

**Figure 4:**
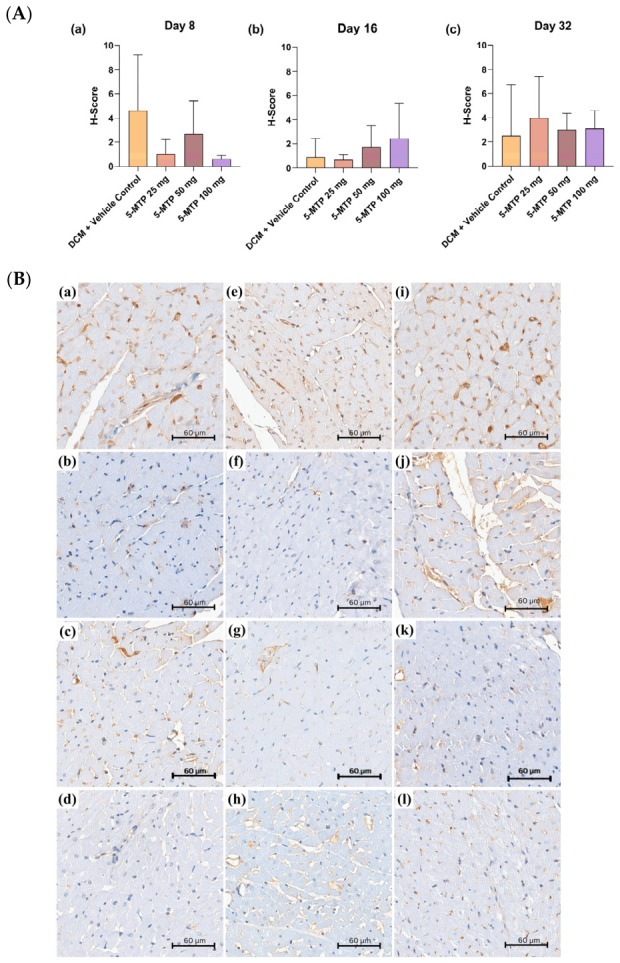
Collagen I immunohistochemical expression across treatment groups at different treatment durations. (**A**) Quantitative comparison of collagen I H-score among treatment groups at day 8 (**a**), day 16 (**b**), and day 32 (**c**). No significant differences in collagen I expression were observed among groups at any time point. (**B**) Representative collagen I immunohistochemical-stained myocardial sections (400× magnification; scale bar = 60 μm) from each treatment group across time points: day 8 DCM + vehicle control (**a**), 5-MTP 25 mg/kg (**b**), 5-MTP 50 mg/kg (**c**), and 5-MTP 100 mg/kg (**d**); day 16 DCM + vehicle control (**e**), 5-MTP 25 mg/kg (**f**), 5-MTP 50 mg/kg (**g**), and 5-MTP 100 mg/kg (**h**); day 32 DCM + vehicle control (**i**), 5-MTP 25 mg/kg (**j**), 5-MTP 50 mg/kg (**k**), and 5-MTP 100 mg/kg (**l**). Brown staining indicates collagen I expression within myocardial tissue. Although early molecular modulation of fibrotic pathways was observed in other markers, collagen I expression did not demonstrate significant between-group differences, suggesting that downstream extracellular matrix deposition may require longer remodeling duration to manifest measurable changes.

For collagen I expression, no significant differences in H-score were observed among groups at any time point (day 8: *p* = 0.556; day 16: *p* = 0.371; day 32: *p* = 0.470) (Figure 4).

### 3.4. Effect of 5-MTP on Cardiomyocyte Apoptosis

**Figure 5:**
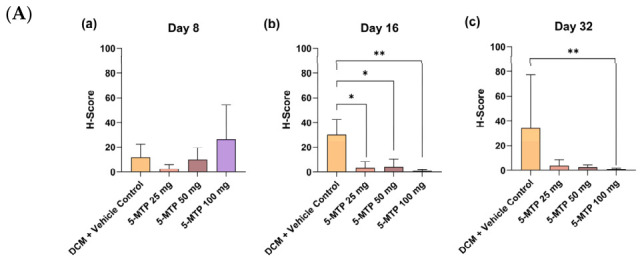

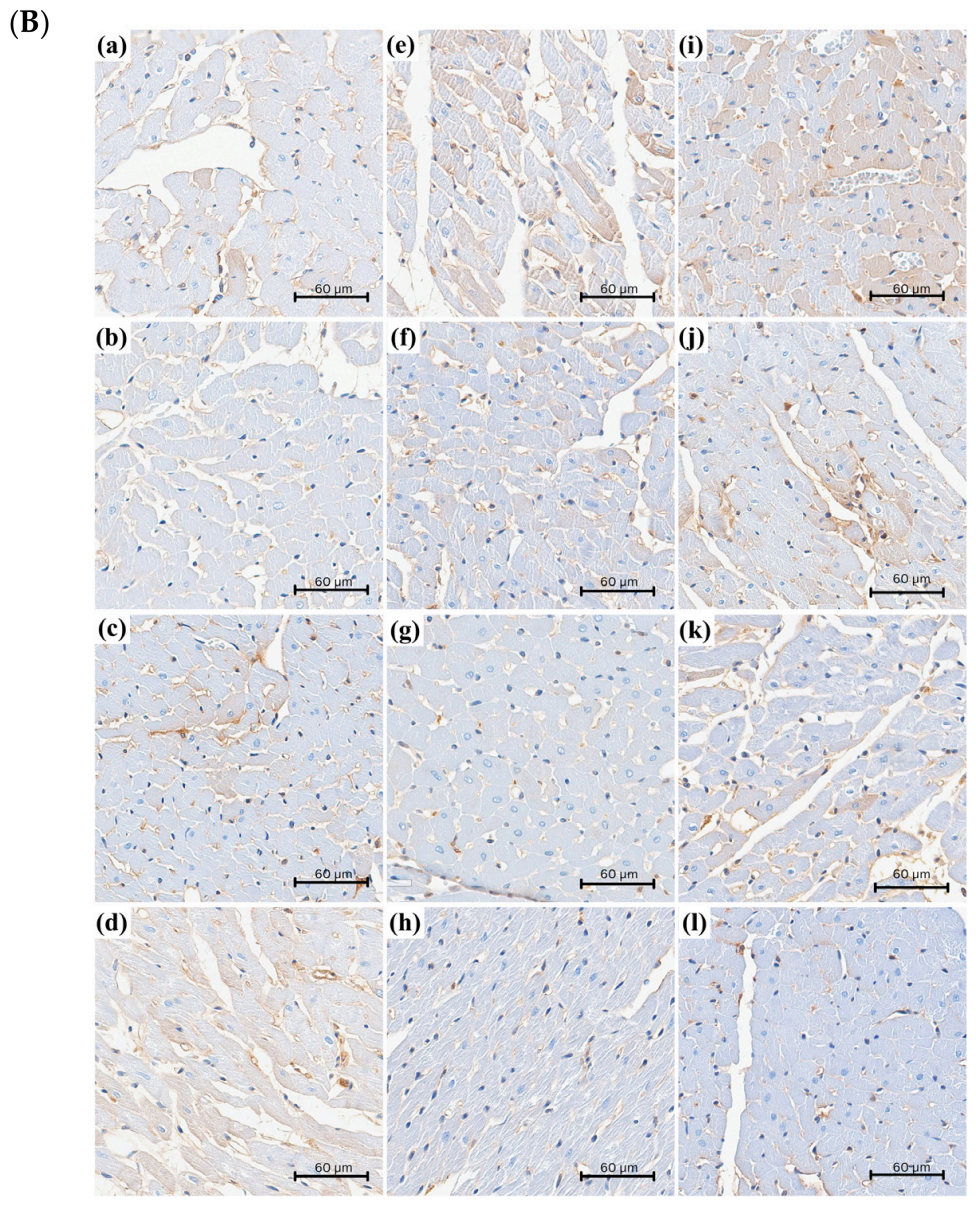
Caspase-3 immunohistochemical expression across treatment groups at different treatment durations. (**A**) Quantitative comparison of caspase-3 H-score among treatment groups at day 8 (**a**), day 16 (**b**), and day 32 (**c**). No significant differences were observed at day 8. At day 16, caspase-3 expression differed significantly among groups, with all 5-MTP treatment groups demonstrating significantly lower H-scores compared with the DCM + vehicle control group (25 mg/kg, *p* = 0.026; 50 mg/kg, *p* = 0.034; 100 mg/kg, *p* = 0.005). At day 32, a significant difference was also observed, with only the 100 mg/kg group showing significant reduction compared with the DCM + vehicle control group (*p* = 0.004). * *p* < 0.05 and ** *p* < 0.01 vs DCM + vehicle control. (**B**) Representative caspase-3 immunohistochemical-stained myocardial sections (400× magnification; scale bar = 60 μm) from each treatment group across time points: day 8 DCM + vehicle control (**a**), 5-MTP 25 mg/kg (**b**), 5-MTP 50 mg/kg (**c**), and 5-MTP 100 mg/kg (**d**); day 16 DCM + vehicle control (**e**), 5-MTP 25 mg/kg (**f**), 5-MTP 50 mg/kg (**g**), and 5-MTP 100 mg/kg (**h**); day 32 DCM + vehicle control (**i**), 5-MTP 25 mg/kg (**j**), 5-MTP 50 mg/kg (**k**), and 5-MTP 100 mg/kg (**l**). Brown staining indicates caspase-3 expression. These findings suggest time-dependent differences in caspase-3 expression following 5-MTP treatment.

In the 8-day treatment duration group, no significant difference in caspase-3 H-score was observed among groups following 5-MTP treatment (*p* = 0.255). However, in the 16-day treatment duration group, a significant difference in caspase-3 expression was observed among groups (*p* = 0.029), with all 5-MTP treatment groups demonstrating significantly lower caspase-3 expression compared with the DCM + vehicle control group (29.1 [15.8–46.4]), including the 25 mg/kg group (1.5 [0–10.4], *p* = 0.026), 50 mg/kg group (1.2 [0.4–13.4], *p* = 0.034), and 100 mg/kg group (0.6 [0–2.0], *p* = 0.005). Additionally, in the 32-day treatment duration group, a significant difference among groups was observed (*p* = 0.035); however, only the 100 mg/kg group showed a significant difference compared with the DCM + vehicle control group (17.3 [4.8–97.0] vs. 0.4 [0–2.3], *p* = 0.004), whereas the 25 mg/kg and 50 mg/kg groups did not differ significantly from the DCM + vehicle control group (*p* > 0.05) (Figure 5).

### 3.5. Effect of 5-MTP on Inflammation and Fibrotic Signaling

#### 3.5.1. TGF-β

**Figure 6:**
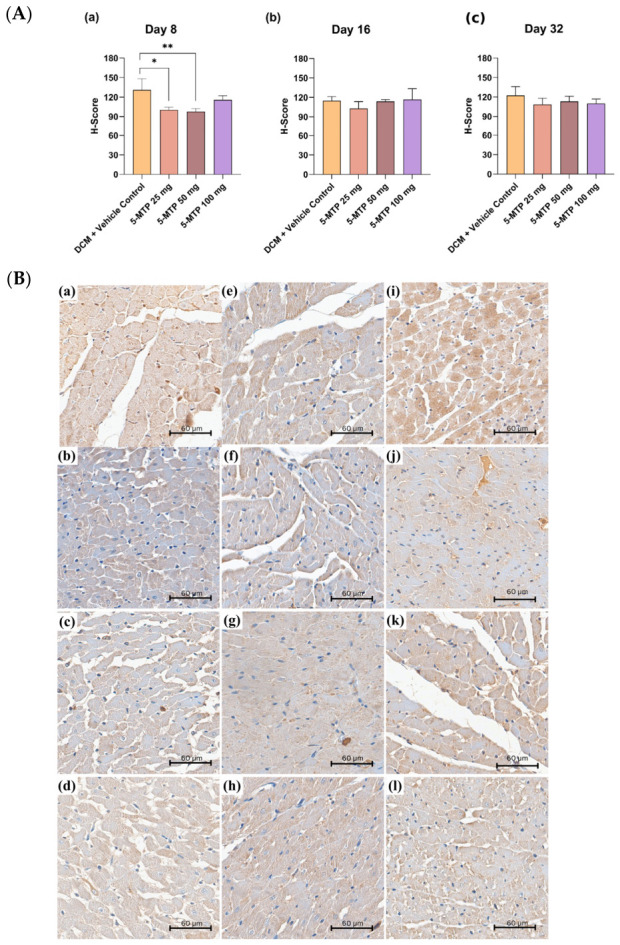
TGF-β immunohistochemical expression across treatment groups at different treatment durations. (**A**) Quantitative comparison of TGF-β H-score among treatment groups at day 8 (**a**), day 16 (**b**), and day 32 (**c**). At day 8, TGF-β expression differed significantly among groups, with the 5-MTP 25 mg/kg (*p* = 0.017) and 50 mg/kg (*p* = 0.002) groups demonstrating significantly lower H-scores compared with the DCM + vehicle control group, while the 100 mg/kg group did not differ significantly. No significant differences were observed at day 16 or day 32. * *p* < 0.05 and ** *p* < 0.01 vs DCM + vehicle control. (**B**) Representative TGF-β immunohistochemical-stained myocardial sections (400× magnification; scale bar = 60 μm) from each treatment group across time points: day 8 DCM + vehicle control (**a**), 5-MTP 25 mg/kg (**b**), 5-MTP 50 mg/kg (**c**), and 5-MTP 100 mg/kg (**d**); day 16 DCM + vehicle control (**e**), 5-MTP 25 mg/kg (**f**), 5-MTP 50 mg/kg (**g**), and 5-MTP 100 mg/kg (**h**); day 32 DCM + vehicle control (**i**), 5-MTP 25 mg/kg (**j**), 5-MTP 50 mg/kg (**k**), and 5-MTP 100 mg/kg (**l**). Brown staining indicates TGF-β expression. These findings suggest 5-MTP was associated with early differences in TGF-β expression, particularly during the initial remodeling phase, before broader downstream fibrotic changes became apparent.

TGF-β expression showed a significant difference among groups at day 8 (*p* = 0.006), with the 25 mg/kg group (99.7 [96.6–105.6], *p* = 0.017) and 50 mg/kg group (96.0 [94.2–104.8], *p* = 0.002) demonstrating significantly lower expression compared with the DCM + vehicle control group (130.1 [113.8–149.6]), whereas no significant difference was observed in the 100 mg/kg group (115.0 [109.8–123.4], *p* = 0.458). However, no significant differences were observed at day 16 (*p* = 0.287) and day 32 (*p* = 0.241) (Figure 6).

#### 3.5.2. SMAD3

**Figure 7:**
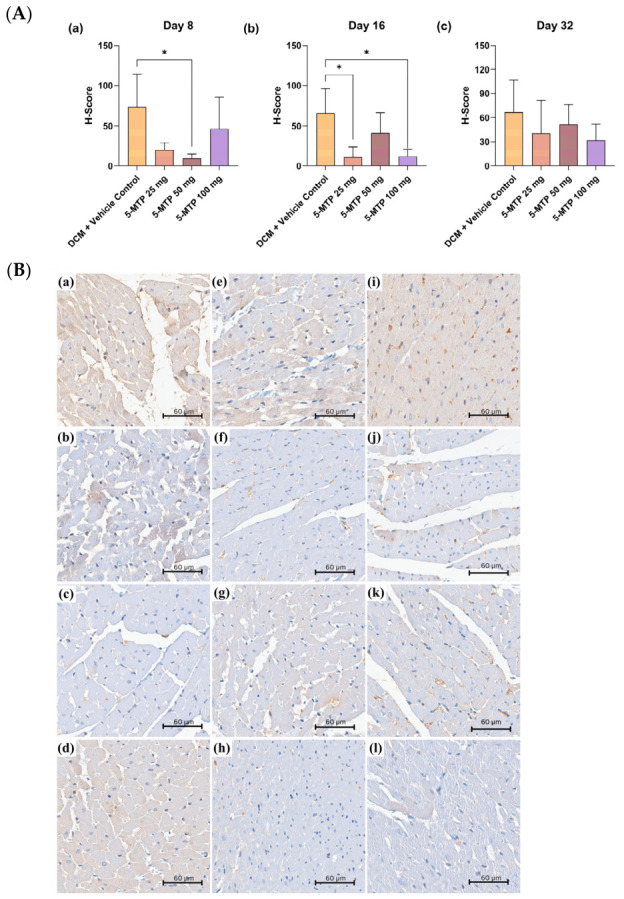
SMAD3 immunohistochemical expression across treatment groups at different treatment durations. (**A**) Quantitative comparison of SMAD3 H-score among treatment groups at day 8 (**a**), day 16 (**b**), and day 32 (**c**). At day 8, SMAD3 expression did show a significant difference in pairwise 5-MTP 50 mg/kg compared to control. At day 16, SMAD3 expression differed significantly among groups, with the 5-MTP 25 mg/kg (*p* = 0.017) and 100 mg/kg (*p* = 0.017) groups demonstrating significantly lower H-scores compared with the DCM + vehicle control group, whereas the 50 mg/kg group did not show a significant difference. No significant differences were observed at day 32. * *p* < 0.05 vs DCM + vehicle control. (**B**) Representative SMAD3 immunohistochemical-stained myocardial sections (400× magnification; scale bar = 60 μm) from each treatment group across time points: day 8 DCM + vehicle control (**a**), 5-MTP 25 mg/kg (**b**), 5-MTP 50 mg/kg (**c**), and 5-MTP 100 mg/kg (**d**); day 16 DCM + vehicle control (**e**), 5-MTP 25 mg/kg (**f**), 5-MTP 50 mg/kg (**g**), and 5-MTP 100 mg/kg (**h**); day 32 DCM + vehicle control (**i**), 5-MTP 25 mg/kg (**j**), 5-MTP 50 mg/kg (**k**), and 5-MTP 100 mg/kg (**l**). Brown staining indicates SMAD3 expression. These findings suggest 5-MTP was associated with intermediate-phase differences in SMAD3 expression, consistent with temporal suppression of active myocardial fibrogenesis during the remodeling process.

SMAD3 expression did not differ significantly among groups at day 8 (*p* = 0.069), although the 50 mg/kg group demonstrated a significant difference compared with the DCM + vehicle control group (8.9 [3.8–16.4] vs. 88.1 [14.0–105.0], *p* = 0.012). At day 16, a significant difference in SMAD3 expression was observed among groups (*p* = 0.029), with the 25 mg/kg group (7.2 [0.2–29.0], *p* = 0.017) and 100 mg/kg group (9.5 [5.2–25.2], *p* = 0.017) demonstrating significantly lower expression compared with the DCM + vehicle control group (72.8 [25.8–91.2]), whereas no significant difference was observed in the 50 mg/kg group (43.6 [12.8–64.4], *p* = 0.552). No significant differences were observed at day 32 (*p* = 0.500) (Figure 7).

#### 3.5.3. AKT

**Figure 8:**
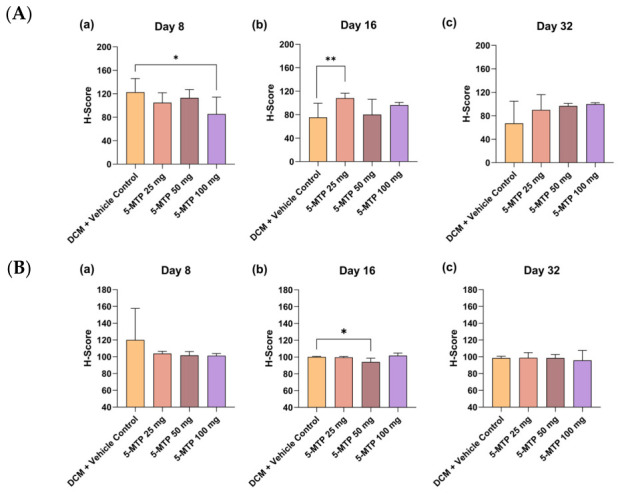

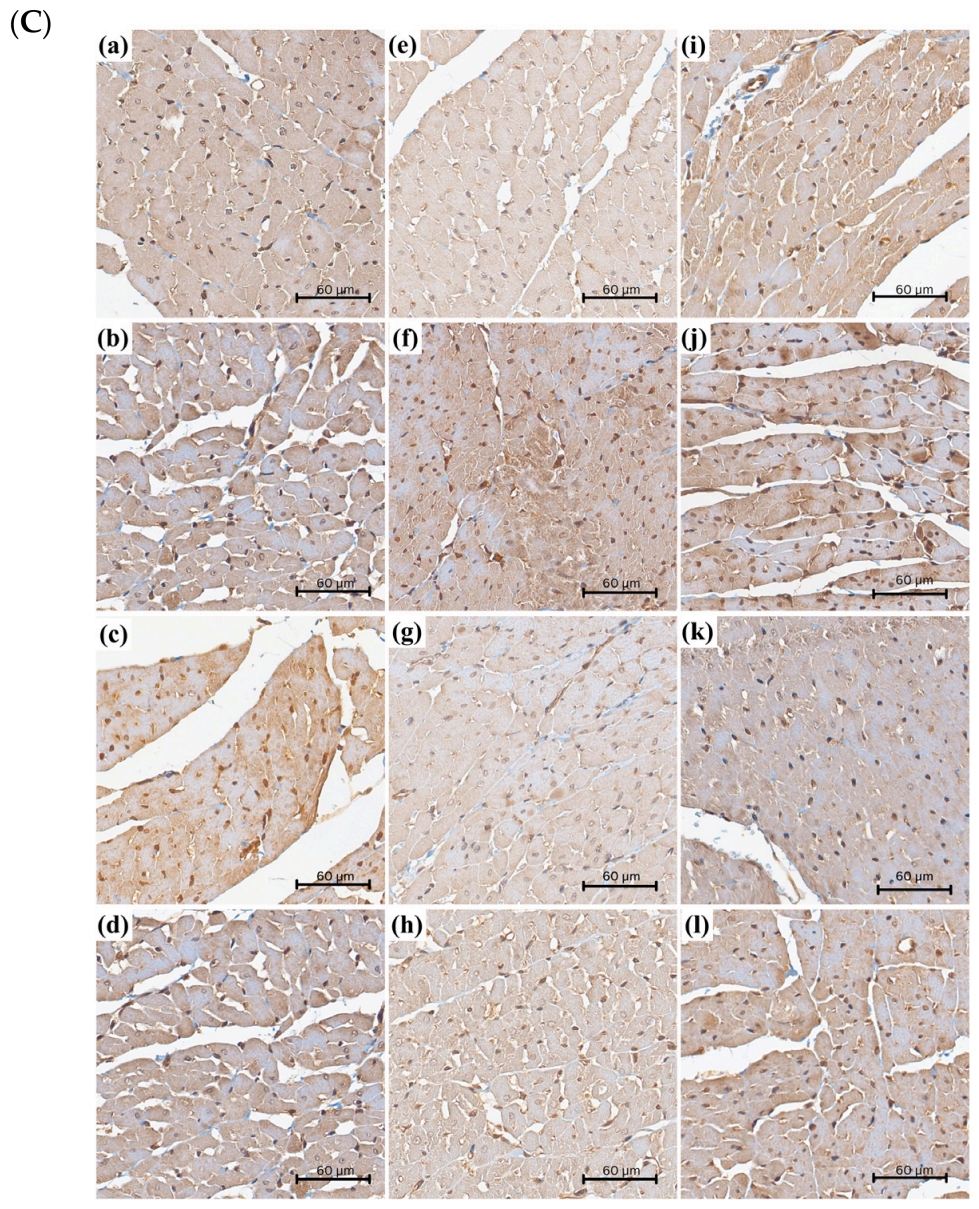
Nuclear and cytoplasmic AKT immunohistochemical expression across treatment groups at different treatment durations. (**A**) Quantitative comparison of AKT nuclear H-score among treatment groups at day 8 (**a**), day 16 (**b**), and day 32 (**c**). At day 8, no significant overall difference was observed among groups (*p* = 0.147), although the 100 mg/kg group demonstrated significantly lower nuclear AKT expression compared with the DCM + vehicle control group (*p* = 0.031). At day 16, AKT nuclear expression differed significantly among groups (*p* = 0.021), with the 25 mg/kg group demonstrating significantly higher nuclear AKT expression compared with the DCM + vehicle control group (*p* = 0.003), whereas no significant differences were observed in the 50 mg/kg or 100 mg/kg groups. No significant differences were observed at day 32. * *p* < 0.05 and ** *p* < 0.01 vs DCM + vehicle control. (**B**) Quantitative comparison of AKT cytoplasmic H-score among treatment groups at day 8 (**a**), day 16 (**b**), and day 32 (**c**). No significant differences were observed at day 8 or day 32. At day 16, AKT cytoplasmic expression differed significantly among groups (*p* = 0.023), with the 50 mg/kg group demonstrating significantly lower cytoplasmic AKT expression compared with the DCM + vehicle control group (*p* = 0.019), while no significant differences were observed in the 25 mg/kg or 100 mg/kg groups. (**C**) Representative AKT immunohistochemical-stained myocardial sections (400× magnification; scale bar = 60 μm) from each treatment group across time points: day 8 DCM + vehicle control (**a**), 5-MTP 25 mg/kg (**b**), 5-MTP 50 mg/kg (**c**), and 5-MTP 100 mg/kg (**d**); day 16 DCM + vehicle control (**e**), 5-MTP 25 mg/kg (**f**), 5-MTP 50 mg/kg (**g**), and 5-MTP 100 mg/kg (**h**); day 32 DCM + vehicle control (**i**), 5-MTP 25 mg/kg (**j**), 5-MTP 50 mg/kg (**k**), and 5-MTP 100 mg/kg (**l**). Brown staining indicates AKT expression. These findings demonstrate time-dependent differences in AKT expression following 5-MTP treatment.

AKT expression was evaluated separately according to nuclear and cytoplasmic localization. For AKT nuclear expression, no significant overall difference was observed among groups at day 8 (*p* = 0.147), although the 100 mg/kg group demonstrated significantly lower expression compared with the DCM + vehicle control group (85.5 ± 28.7 vs. 122.7 ± 23.3, *p* = 0.031). At day 16, AKT nuclear expression differed significantly among groups (*p* = 0.021), with the 25 mg/kg group demonstrating significantly higher expression compared with the DCM + vehicle control group (106.0 [101.2–119.6] vs. 80.5 [42.0–98.2], *p* = 0.003), whereas no significant differences were observed in the 50 mg/kg or 100 mg/kg groups. No significant overall difference in AKT nuclear expression was observed at day 32 (*p* = 0.314) (Figure 8).

For AKT cytoplasmic expression, no significant overall differences were observed at day 8 (*p* = 0.564) or day 32 (*p* = 0.914). However, at day 16, AKT cytoplasmic expression differed significantly among groups (*p* = 0.023), with the 50 mg/kg group demonstrating significantly lower expression compared with the DCM + vehicle control group (95.2 [88.2–98.0] vs. 100.0 [99.8–101.0], *p* = 0.019), whereas no significant differences were observed in the 25 mg/kg or 100 mg/kg groups (Figure 8).

#### 3.5.4. NF-κB

**Figure 9:**
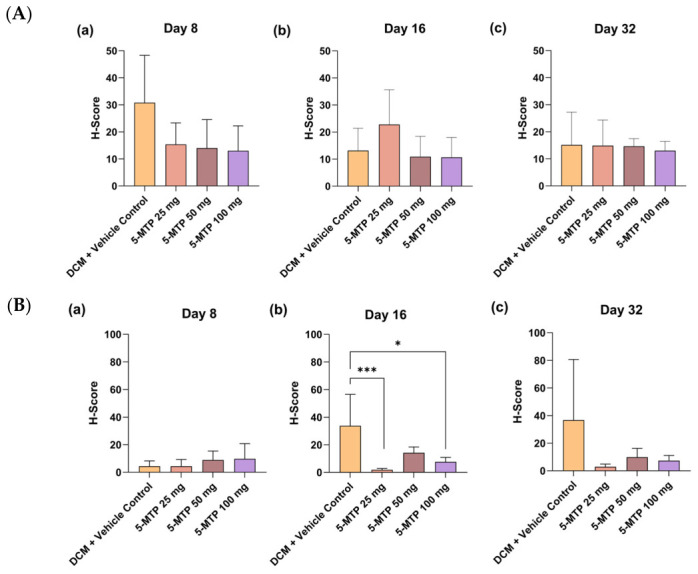

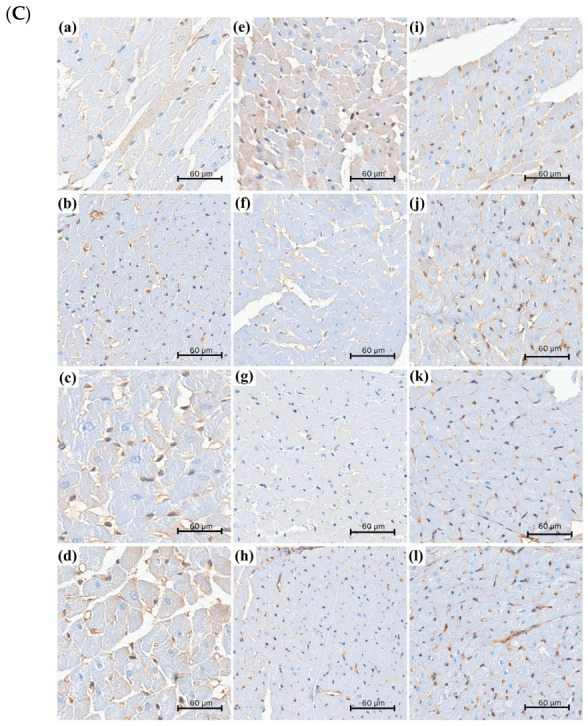
Nuclear and cytoplasmic NF-κB immunohistochemical expression across treatment groups at different treatment durations. (**A**) Quantitative comparison of NF-κB nuclear H-score among treatment groups at day 8 (**a**), day 16 (**b**), and day 32 (**c**). No significant differences in nuclear NF-κB expression were observed among groups at any time point. (**B**) Quantitative comparison of NF-κB cytoplasmic H-score among treatment groups at day 8 (**a**), day 16 (**b**), and day 32 (**c**). No significant differences were observed at day 8 or day 32. At day 16, cytoplasmic NF-κB expression differed significantly among groups (*p* = 0.006), with the 5-MTP 25 mg/kg group demonstrating significantly lower expression compared with the DCM + vehicle control group (*p* < 0.001), and the 100 mg/kg group also demonstrating significantly lower expression (*p* = 0.045), whereas no significant difference was observed in the 50 mg/kg group. * *p* < 0.05 and *** *p* < 0.001 vs DCM + vehicle control. (**C**) Representative NF-κB immunohistochemical-stained myocardial sections (400× magnification; scale bar = 60 μm) from each treatment group across time points: day 8 DCM + vehicle control (**a**), 5-MTP 25 mg/kg (**b**), 5-MTP 50 mg/kg (**c**), and 5-MTP 100 mg/kg (**d**); day 16 DCM + vehicle control (**e**), 5-MTP 25 mg/kg (**f**), 5-MTP 50 mg/kg (**g**), and 5-MTP 100 mg/kg (**h**); day 32 DCM + vehicle control (**i**), 5-MTP 25 mg/kg (**j**), 5-MTP 50 mg/kg (**k**), and 5-MTP 100 mg/kg (**l**). Brown staining indicates NF-κB expression. These findings indicate differences in cytoplasmic NF-κB expression at the intermediate time point following 5-MTP treatment.

Nuclear NF-κB expression did not differ significantly among groups at any time point, including day 8 (*p* = 0.375), day 16 (*p* = 0.259), and day 32 (*p* = 0.981). Cytoplasmic NF-κB expression showed no significant difference at day 8 (*p* = 0.702) or day 32 (*p* = 0.220). However, a significant difference in cytoplasmic NF-κB expression was observed at day 16 (*p* = 0.006), with the 25 mg/kg group (2.0 [0.6–3.0], *p* < 0.001) and 100 mg/kg group (8.6 [3.2–10.0], *p* = 0.045) demonstrating significantly lower expression compared with the DCM + vehicle control group (28.5 [12.0–65.8]), whereas no significant difference was observed in the 50 mg/kg group (14.4 [9.2–18.7], *p* = 0.373) (Figure 9).

A comprehensive summary of temporal histopathological and molecular changes across all assessed parameters is presented in Table 1.

### 3.6. MANOVA Analysis

MANOVA demonstrated a significant simultaneous effect of treatment group on fibrosis-related molecular parameters at day 8 (Wilks’ Lambda = 0.072, F = 5.289, *p* < 0.001), with a partial eta squared of 0.584, indicating that 58.4% of the variance in combined fibrosis parameters was explained by treatment group. Follow-up univariate analysis showed that TGF-β (partial η^2^ = 0.714, *p* = 0.001) and SMAD3 (partial η^2^ = 0.502, *p* = 0.034) differed significantly among groups, whereas AKT nuclear expression did not show a significant difference (partial η^2^ = 0.350, *p* = 0.147). Among these parameters, TGF-β demonstrated the largest effect size (Table 2).

At day 16, MANOVA also demonstrated a significant simultaneous effect of treatment group on combined cardiomyopathy-related parameters (Wilks’ Lambda = 0.007, F = 5.363, *p* < 0.001), with a partial eta squared of 0.808, indicating that 80.8% of the variance was explained by treatment group. Follow-up univariate analysis demonstrated significant differences in caspase-3 (partial η^2^ = 0.774, *p* < 0.001), NF-κB cytoplasmic expression (partial η^2^ = 0.583, *p* = 0.012), SMAD3 (partial η^2^ = 0.603, *p* = 0.009), AKT cytoplasmic expression (partial η^2^ = 0.576, *p* = 0.014), and Masson’s trichrome fibrosis score (partial η^2^ = 0.545, *p* = 0.020), whereas AKT nuclear expression did not differ significantly (partial η^2^ = 0.402, *p* = 0.094). Caspase-3 demonstrated the largest effect size among all evaluated parameters. Detailed univariate MANOVA results are provided in Supplementary Table S6.

## 4. Discussion

The main findings of this in vivo experimental study were as follows: (1) treatment with 5-MTP did not significantly affect myocardial inflammatory histopathology at any time point; (2) 5-MTP significantly modulated myocardial fibrosis at the intermediate time point, as reflected by differences in Masson’s trichrome staining, although no significant changes were observed in collagen I expression; (3) 5-MTP significantly modulated cardiomyocyte apoptosis at the intermediate time point, as indicated by differences in caspase-3 expression; and (4) 5-MTP demonstrated time-dependent changes in inflammatory and fibrotic related molecular markers, with early change of TGF-β expression and subsequent alterations in SMAD3, AKT, and cytoplasmic NF-κB expression at the intermediate time point.

An endogenous metabolite of tryptophan, 5-MTP has been reported to exert cardioprotective effects through anti-inflammatory, anti-apoptotic, and anti-fibrotic mechanisms [6,7]. These pleiotropic effects are particularly relevant in diabetic cardiomyopathy, where persistent hyperglycemia, oxidative stress, and advanced glycation end-products (AGEs) contribute to myocardial injury through activation of inflammatory signaling, induction of cardiomyocyte apoptosis, and progressive extracellular matrix remodeling [9]. Therefore, modulation of these interconnected pathways represents a potential therapeutic approach in attenuating myocardial damage in DCM.

Hematoxylin–eosin staining is a standard histopathological method used to evaluate myocardial tissue morphology and inflammatory cell infiltration, including lymphocytes, macrophages, and neutrophils [10]. In the present study, myocardial inflammatory scores did not differ significantly among groups at any time point. This finding may reflect the early phase of diabetic cardiomyopathy in this model, in which inflammatory activation may not yet be sufficiently advanced to produce detectable histopathological changes. However, the observed differences in cytoplasmic NF-κB expression at the intermediate time point suggest that inflammatory modulation may have occurred at the molecular level prior to the development of overt histopathological changes. NF-κB is a key regulator of inflammatory responses in diabetic cardiomyopathy [11]. Increased NF-κB expression has been shown to activate TLR4/NLRP3 inflammasome signaling, leading to enhanced pro-inflammatory cytokine production, oxidative stress, and myocardial remodeling, as reported by Saleh et al. [12]. Therefore, the absence of significant findings on HE staining does not exclude the possibility of alterations in inflammation-related molecular markers following 5-MTP treatment. Nevertheless, these findings should be interpreted cautiously, as additional inflammatory mediators and oxidative stress markers were not evaluated in the present study. Further studies incorporating a broader panel of inflammatory markers are required to better characterize the potential effects of 5-MTP on inflammation-related signaling in diabetic cardiomyopathy.

The antifibrotic effect of 5-MTP was demonstrated by the significant difference in myocardial fibrosis observed at the intermediate time point using Masson’s trichrome staining. Masson’s trichrome provides a structural assessment of collagen deposition and extracellular matrix remodeling. Consistent with previous findings, Shuai et al. reported that 5-MTP reduced myocardial fibrosis and collagen deposition in experimental models [13]. However, collagen I expression did not differ significantly among groups in this study. This discrepancy may be explained by the temporal progression of fibrotic remodeling, in which modulation of upstream signaling pathways precedes measurable changes in collagen deposition. In addition, myocardial fibrosis is a complex process involving multiple extracellular matrix components, fibroblast activity, and tissue architecture, rather than being solely determined by collagen I expression. Collagen I IHC may reflect specific ECM protein abundance, whereas MT captures broader architectural collagen deposition and interstitial remodeling. Therefore, the observed antifibrotic findings should be interpreted cautiously.

The observed changes in TGF-β and SMAD3 expression are consistent with the involvement of upstream fibrotic signaling pathways. TGF-β is a key mediator of fibrogenesis in diabetic cardiomyopathy, acting through activation of SMAD-dependent pathways that promote fibroblast activation and extracellular matrix deposition [14,15]. In this study, TGF-β expression was altered at the early time point, whereas SMAD3 expression differed at the intermediate time point, suggesting a temporal cascade from upstream to downstream signaling. Similar findings have been reported by Hsu et al., who demonstrated that 5-MTP suppresses TGF-β expression and reduces fibrogenesis [16]. In addition, Fang et al. showed that 5-MTP inhibits SMAD3 activation by suppressing SMAD3 phosphorylation, thereby reducing fibroblast-to-myofibroblast differentiation and extracellular matrix accumulation [7].

Caspase-3 is a key effector of apoptosis in cardiomyocytes. In diabetic cardiomyopathy, persistent hyperglycemia and advanced glycation end-products promote oxidative stress and mitochondrial dysfunction, leading to activation of the intrinsic apoptotic pathway [17,18]. The significant difference in caspase-3 expression at the intermediate time point may reflect alterations in apoptosis-related molecular markers following 5-MTP treatment, suggesting that 5-MTP may attenuate cardiomyocyte apoptosis during active disease progression. This is consistent with findings by Hsu et al., who demonstrated that 5-MTP reduces cardiomyocyte apoptosis, decreases caspase-3 expression, and improves myocardial injury through attenuation of oxidative stress and stabilization of mitochondrial function [16].

Changes in AKT expression may be associated with the observed alterations in apoptosis-related markers. AKT is a key component of the PI3K/AKT survival pathway and plays an important role in cardiomyocyte survival, anti-apoptotic signaling, and adaptation to metabolic stress [19,20]. The significant difference in AKT expression at the intermediate time point may reflect activation of a survival response during myocardial injury. Previous work by Shiraishi et al. [21] demonstrated that nuclear-targeted AKT enhances cardiomyocyte survival by inhibiting caspase activation and reducing DNA fragmentation, supporting the role of AKT in cardiomyocyte survival signaling. In addition, AKT may also interact with fibrotic signaling pathways downstream of TGF-β, highlighting its role as a cross-regulatory signaling mediator.

Collectively, these findings suggest that the effects of 5-MTP in diabetic cardiomyopathy are time-dependent and pathway-specific. Molecular changes in inflammation and fibrosis-related markers, including NF-κB, TGF-β, SMAD3, and AKT, appears to occur earlier than detectable structural changes on histopathological examination or collagen I expression. This supports the concept that 5-MTP primarily acts at the upstream molecular level before translating into more stable structural remodeling.

**Figure 10:**
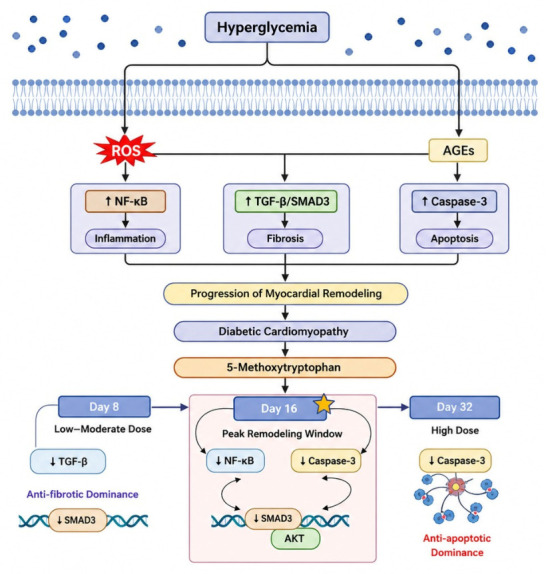
Proposed temporal framework of observed histopathological and molecular changes following 5-MTP treatment in experimental diabetic cardiomyopathy. Hyperglycemia promotes ROS and AGE formation, activating NF-κB-mediated inflammation, TGF-β/SMAD3-driven fibrosis, and caspase-3-associated apoptosis, which contribute to myocardial remodeling progression. Based on this study, 5-MTP was associated with early changes in TGF-β expression (day 8), broader intermediate changes in NF-κB, SMAD3, AKT, and caspase-3 expression (day 16), and persistent differences in caspase-3 expression at day 32. This figure summarizes the proposed temporal molecular effects of 5-MTP during DCM progression.

The MANOVA findings further support the time-dependent effects of 5-MTP on myocardial remodeling in diabetic cardiomyopathy. At the early phase, observed differences were primarily concentrated on fibrosis-related molecular signaling, as demonstrated by significant multivariate effects at day 8 predominantly driven by TGF-β and SMAD3. The dominant effect of TGF-β at this stage suggests that 5-MTP may initially act through modulation of upstream profibrotic pathways before broader structural or downstream cellular changes become evident. This pattern is consistent with the concept that molecular alterations in fibrotic signaling may precede measurable histopathological remodeling. Collectively, these findings suggest that 5-MTP exerts temporally staged effects on myocardial remodeling, with early changes in upstream profibrotic pathways, broader intermediate effects on inflammatory, fibrotic, and survival signaling, and later persistence of apoptosis-related differences (Figure 10).

Several limitations should be considered. First, the limited sample size per group may have reduced statistical power to detect modest treatment effects, additionally, the absence of healthy control group without DCM also limits the analysis and conclusion that could be drawn. Consequently, some observed differences may reflect biological variability rather than true treatment effects, whereas modest treatment effects may have remained undetected. In addition, the small sample size may have limited the robustness of the statistical analyses. Therefore, the findings should be interpreted as exploratory and hypothesis-generating. Larger studies are needed to confirm the observed effects. Second, the relatively short follow-up of 32 days may have precluded observation of delayed functional benefits, as myocardial remodeling in diabetic cardiomyopathy is a chronic and progressive process. Changes in inflammation, apoptosis, and fibrosis may not have been fully captured within this timeframe. Third, dose optimization may be required to enhance treatment response and potentially sustain the early beneficial structural effects observed in our study, which appeared attenuated at later follow-up. In addition, molecular assessments were based exclusively on immunohistochemical analyses. Although IHC provides information regarding tissue-level protein expression and localization, it does not establish direct pathway activation or inhibition. Therefore, the proposed mechanistic framework should be considered exploratory and hypothesis-generating. Further studies incorporating Western blotting, quantitative PCR, phosphorylation analyses, and pathway-specific functional experiments are required to confirm the underlying molecular mechanisms. Furthermore, a concurrent non-diabetic control group was not included in the intervention. Therefore, the extent to which 5-MTP restored pathological markers toward physiological levels could not be directly determined.

## 5. Conclusions

In conclusion, 5-MTP was associated with time-dependent changes in myocardial remodeling-related histopathological and molecular markers in experimental DCM. These effects were most evident at the molecular level, particularly in the expression of NF-κB, TGF-β/SMAD3, AKT, and caspase-3 pathways, with corresponding differences in myocardial fibrosis assessed by Masson’s trichrome staining. Given the exploratory nature of this study and the absence of biochemical and functional validation, these findings should be considered hypothesis-generating and warrant further investigation in larger studies to clarify the potential role of 5-MTP in diabetic cardiomyopathy.

## Figures and Tables

**Table 1:** Summary of temporal effects of 5-MTP on myocardial inflammation, apoptosis, fibrosis, and related molecular pathways in experimental diabetic cardiomyopathy.

Outcome	Day 8	Day 16	Day 32
HE	NS (overall *p* = 0.366)	NS (overall *p* = 0.642)	NS (overall *p* = 0.727)
MT	NS (overall *p* = 0.246)	Significant difference (overall *p* = 0.020 *) DCM 46.6 ± 2.7 25 mg 35.3 ± 4.3 (*p* = 0.005 *) 50 mg 36.1 ± 3.5 (*p* = 0.008 *) 100 mg 38.8 ± 7.1 (*p* = 0.037 *)	NS (overall *p* = 0.367)
Collagen I	NS (overall *p* = 0.556)	NS (overall *p* = 0.371)	NS (overall *p* = 0.470)
Caspase-3	NS (overall *p* = 0.255)	Significant difference (overall *p* = 0.029 *) DCM 29.1 (15.8–46.4) 25 mg 1.5 (0–10.4), *p* = 0.026 * 50 mg 1.2 (0.4–13.4), *p* = 0.034 * 100 mg 0.6 (0–2.0), *p* = 0.005 *	Significant difference (overall *p* = 0.035 *) DCM 17.3 (4.8–97.0); 25 mg 2.1 (0–10.4), *p* = 0.053 50 mg 2.3 (0.8–4.6), *p* = 0.080 100 mg 0.4 (0–2.3), *p* = 0.004 *
TGF-β	Significant difference (overall *p* = 0.006 *) DCM 130.1 (113.8–149.6) 25 mg 99.7 (96.6–105.6), *p* = 0.017 * 50 mg 96.0 (94.2–104.8), *p* = 0.002 * 100 mg 115.0 (109.8–123.4), *p* = 0.458	NS (overall *p* = 0.287)	NS (overall *p* = 0.241)
SMAD3	NS (overall *p* = 0.069)	Significant difference (overall *p* = 0.029 *) DCM 72.8 (25.8–91.2) 25 mg 7.2 (0.2–29.0), *p* = 0.017 * 50 mg 43.6 (12.8–64.4), *p* = 0.552 100 mg 9.5 (5.2–25.2), *p* = 0.017 *	NS (overall *p* = 0.500)
AKT nuclear	NS (overall *p* = 0.147)	Significant difference (overall *p* = 0.021 *) DCM 80.5 (42.0–98.2) 25 mg 106.0 (101.2–119.6), *p* = 0.003 * 50 mg 87.2 (45.4–100.6), *p* = 0.480 100 mg 97.4 (90.4–100.0), *p* = 0.281	NS (overall *p* = 0.314)
AKT cytoplasmic	NS (overall *p* = 0.564)	Significant difference (overall *p* = 0.023 *) DCM 100.0 (99.8–101.0) 25 mg 99.9 (98.0–100.4), *p* = 0.624 50 mg 95.2 (88.2–98.0), *p* = 0.019 * 100 mg 100.1 (100.0–106.4), *p* = 0.571	NS (overall *p* = 0.914)
NF-κB nuclear	NS (overall *p* = 0.375)	NS (overall *p* = 0.259)	NS (overall *p* = 0.981)
NF-κB cytoplasmic	NS (overall *p* = 0.702)	Significant difference (overall *p* = 0.006 *) DCM 28.5 (12.0–65.8) 25 mg 2.0 (0.6–3.0), *p* < 0.001 * 50 mg 14.4 (9.2–18.7) 100 mg 8.6 (3.2–10.0), *p* = 0.045 *	NS (overall *p* = 0.220)

NS = not significant. Values are presented as mean ± SD or median (minimum–maximum), as appropriate. Overall group differences were analyzed using one-way ANOVA or Kruskal–Wallis test, followed by post hoc pairwise comparisons when applicable. * *p* < 0.05 was considered statistically significant.

**Table 2:** MANOVA summary of treatment effects on fibrosis- and remodeling-related parameters.

Time Point	Wilks’ Lambda	F	Partial η^2^	Overall *p*-Value	Dominant Parameters
Day 8	0.072	5.289	0.584	<0.001	TGF-β, SMAD3
Day 16	0.007	5.363	0.808	<0.001	Caspase-3, cytoplasmic NF-κB, SMAD3, cytoplasmic AKT, MT

MANOVA was performed separately at each time point. Partial η^2^ represents effect size of treatment group on combined parameters.

## Data Availability

The data presented in this study are available upon reasonable request from the corresponding author.

## Supplementary Materials

The following supporting information can be downloaded at: https://www.mdpi.com/article/10.3390/life16071198/s1.

## Abbreviations

The following abbreviations are used in this manuscript:

5-MTP	5-Methoxytryptophan
AGEs	Advanced Glycation End-products
AKT	Protein Kinase B
ANOVA	Analysis of Variance
DAB	Diaminobenzidine
DCM	Diabetic Cardiomyopathy
DM	Diabetes Mellitus
ECM	Extracellular Matrix
EDTA	Ethylenediaminetetraacetic Acid
HE	Hematoxylin and Eosin
HFHF	High-Fat High-Fructose
H-score	Histological Score
IHC	Immunohistochemistry/Immunohistochemical
IQR	Interquartile Range
MANOVA	Multivariate Analysis of Variance
MT	Masson’s Trichrome
NF-κB	Nuclear Factor Kappa B
PBS	Phosphate-Buffered Saline
PI3K	Phosphoinositide 3-Kinase
SD	Standard Deviation
SMAD3	Mothers Against Decapentaplegic Homolog 3
STZ	Streptozotocin
TGF-β	Transforming Growth Factor Beta
TLR4	Toll-Like Receptor 4
