# Supplementary material for: Protective Effects of 5-MTP in a Rat Model of Diabetic Cardiomyopathy Through Anti-Inflammatory, Anti-Apoptotic, and Antifibrotic Mechanisms
Source: Life (Basel). 2026 Jul 20;16(7):1198. doi: 10.3390/life16071198 (PMC13413049; doi:10.3390/life16071198)
Supplement: Supplementary file 1 [file life-16-01198-s001.zip › life-4366061-supplementary.pdf]

Supplementary Materials

Supplementary S1. Procedural details of the *in vivo* experimental research

Experimental Animal Housing

Rats were housed in a dedicated animal maintenance room at the Animal Research Facility under controlled environmental conditions, including a temperature of 24–26°C, relative humidity of 65–75%, continuous air circulation with an exhaust fan, and a 12-hour light/12-hour dark artificial lighting cycle. Animals were kept in communal polypropylene cages (50 cm × 40 cm × 20 cm) fitted with stainless steel grid covers and wood shavings as bedding material. Each cage housed two rats. Cages were cleaned and bedding was replaced every 3 days. Body weight was measured upon arrival and subsequently monitored weekly.

Normal Diet

A normal diet was provided to all groups during the acclimatization period. Standard chow and drinking water were available *ad libitum*, with an estimated dietary intake of 20–30 g per rat per day. Food was placed on the wire cage cover, while drinking water was supplied in bottles attached to the cage cover. Food and fluid intake were recorded and measured daily. Food intake was calculated as the difference between the amount of food provided on the previous day and the remaining food prior to replenishment each morning.

High-Fat High-Fructose (HFHF) Diet

Following the acclimatization period, a high-fat high-fructose (HFHF) diet was administered until the end of the study to induce diabetic cardiomyopathy. The high-fat diet consisted of pellets containing 458.32 kcal/100 g, composed of 32.48% fat, 10.70% protein, 51.05% carbohydrates, 10.09% moisture, and 4.68% ash, obtained from Universitas Brawijaya, Malang, Indonesia.

The high-fructose component was administered as a 55% fructose solution at a dose of 1.5 mL via intragastric gavage twice daily. The use of the HFHF diet was intended to accelerate myocardial tissue injury, thereby enabling detailed investigation of the morphological, biochemical, and functional characteristics underlying the pathogenesis of cardiovascular abnormalities.

Blood Glucose Measurement

Blood glucose levels were measured using an enzymatic method after rats were fasted for 6–8 hours. Fasting blood glucose was assessed weekly throughout the study until its completion, including prior to initiation of the high-fat high-fructose (HFHF) diet to obtain baseline glucose levels, prior to 5-MTP treatment, and at the end of the treatment period before euthanasia. Blood samples were obtained from the lateral tail vein, and blood glucose levels were measured using a glucometer.

Measurement of NT-proBNP Levels

Serum NT-proBNP levels were measured using a commercially available ELISA kit (Elabscience®, E-EL-R3023, Wuhan, China) according to the manufacturer's instructions. Briefly, serum samples and ELISA reagents were removed from refrigerated storage and equilibrated to room temperature. Samples were centrifuged (spin-down) at 8,000 rpm for 2 minutes, after which standard solutions and test samples were prepared.

A total of 100 µL of standards or samples was added to each well and incubated for 90 minutes at 37°C. The liquid was then discarded, followed immediately by addition of 100 µL biotinylated detection antibody solution to each well, with incubation for 60 minutes at 37°C. After incubation, the plate was aspirated and washed three times. Subsequently, 100 µL of streptavidin-HRP working solution was added to each well and incubated for 30 minutes at 37°C. The plate was then aspirated and washed five times.

Next, 90 μL of TMB substrate solution was added to each well and incubated for 15 minutes at 37°C. The reaction was terminated by adding 50 μL stop solution. Absorbance was measured immediately at 450 nm, and NT

-proBNP concentrations were calculated according to the standard curve.

Histopathological Examination

Myocardial tissues were harvested from rat hearts, rinsed with distilled water, and fixed in 10% neutral buffered formalin. The samples were subsequently embedded in paraffin and sectioned at a thickness of 5 μm. The sections were stained with hematoxylin and eosin (HE) and Masson's trichrome (MT) and examined under a light microscope.

H&E-stained sections were evaluated semi-quantitatively for myocardial structural alterations, including cardiomyocyte degeneration, cardiomyocyte hypertrophy, myocardial disarray, fatty infiltration, and inflammatory cell infiltration. Each parameter was graded using a four-point scale: 0 (normal), 1 (mild), 2 (moderate), and 3 (severe). A total histopathological score was calculated by summing all parameter scores, representing the overall degree of myocardial injury. (doi:10.3390/ani15050703)

MT-stained sections were analyzed to assess myocardial fibrosis and collagen deposition. Five randomly selected microscopic fields from each sample were evaluated, and the percentage of fibrotic area was determined for each field. The mean fibrosis percentage was subsequently calculated for each sample. (doi:10.1007/s00210-023-02921-8)

Histopathological assessments were performed by observers blinded to treatment allocation.

Immunohistochemical studies

Immunohistochemical staining was performed to evaluate the expression of inflammatory, apoptotic, and fibrotic markers in myocardial tissue, including nuclear factor kappa B (NF-κB), caspase-3, transforming growth factor-β (TGF-β), SMAD3, pro-tein kinase B (AKT), and collagen I.

Paraffin-embedded myocardial tissue sections (3 μm thickness) were deparaffinized in xylene and rehydrated through graded ethanol solutions. Antigen retrieval was performed using Tris–EDTA buffer (pH 9) at 95°C followed by cooling to room temperature and washing in phosphate-buffered saline (PBS). Endogenous peroxidase activity and nonspecific protein binding were blocked prior to antibody incubation.

Sections were incubated with primary antibodies against NF-κB p65 (1:200, Cell Signaling Technology, #8242), caspase-3 (1:300, Cell Signaling Technology, #9662), TGF-β (1:300, Invitrogen, PA1-29020), AKT (1:250, Abcam, ab179463), SMAD3 (1:300, Invitrogen, 51-1500), and collagen I (1:100, Invitrogen, PA1-26204). Primary antibody incubation was performed for 60 min for NF-κB p65, caspase-3, and TGF-β, and overnight (24 h) for AKT, SMAD3, and collagen I. Sections were subsequently incubated with Novolink™ Post Primary reagent and Novolink™ Polymer Detection System (Leica Biosystems) for 30 min each, followed by visualization using diaminobenzidine (DAB) chromogen. The slides were counterstained with hematoxylin, dehydrated, cleared, and mounted.

The stained sections were scanned using a slide scanner (Leica Aperio GT 450, Leica Biosystems, USA) and analyzed under ×400 magnification. The expression of NF-κB was evaluated in both nuclear and cytoplasmic compartments, caspase-3 and TGF-β in the cytoplasm, AKT in nuclear and cytoplasmic regions, SMAD3 in the cytoplasm, and collagen I in the extracellular matrix, in accordance with antibody datasheets.

Quantification of Immunohistochemical Expression

Protein expression levels were quantified using the H-score method with ImageJ software. The H-score was calculated using the following formula:

H-score = (% weakly stained cells × 1) + (% moderately stained cells × 2) + (% strongly stained cells × 3)

where staining intensity was categorized as weak (1+), moderate (2+), or strong (3+). The average H-score was calculated from five randomly selected high-power fields (×400) within the left ventricular myocardium for each sample.

Immunohistochemical Controls and Detection System

Immunohistochemical staining was performed using the Novolink™ Polymer Detection System (Leica Biosystems; product numbers RE7140-K, RE7150-K, RE7280-K, and RE7290-K). Post-primary reagent and polymer incubation were each performed for 30 min.

Negative controls were included in each staining run by omitting the primary antibody. Positive tissue controls were used for antibody optimization and validation as follows: tonsil tissue for NF-κB p65 and caspase-3, placenta for TGF-β, lung carcinoma tissue for AKT, renal carcinoma tissue for collagen I, and breast carcinoma tissue for SMAD3.

Supplementary Table S1. Fasting blood glucose levels from the pilot model-characterization study of the HFHF/STZ-induced early-stage diabetic cardiomyopathy model.

Group (n=4/group)	FBG post HFHF and STZ		
	**Mean ± SD**	**P-value**	**P-value**
Control	87,3 ± 8,1		0,035*[c]
HFHF + STZ25 mg/kgBW	240,0 ± 67,7	0,008*	1,000[a]
HFHF + STZ 40 mg/kgBW	208,3 ± 64,8		0,010*[b]

[a] HFHF + STZ 25 mg/kgBW vs HFHF + STZ 40 mg/kgBW.

[b] HFHF + STZ 40 mg/kgBW vs Control.

[c] HFHF + STZ 25 mg/kgBW vs Control.

Supplementary Table S2. Serum NT-proBNP levels from the pilot model-characterization study of the HFHF/STZ-induced early-stage diabetic cardiomyopathy model.

Group (n=4/group)	NT-proBNP Levels		
	**Mean ± SD (n = 12)**	**P-value**	**P-value**
Control	624,3 ± 126,4	0,001*	0,018*[a]
HFHF + STZ25 mg/kgBW	1121,7 ± 222,5		0,001*[b]
HFHF + STZ 40 mg/kgBW	1367,6 ± 225,0		0,332[c]

[a] HFHF + STZ 25 mg/kgBW vs Control.

[b] HFHF + STZ 40 mg/kgBW vs Control.

[c] HFHF + STZ 25 mg/kgBW vs HFHF + STZ 40 mg/kgBW.

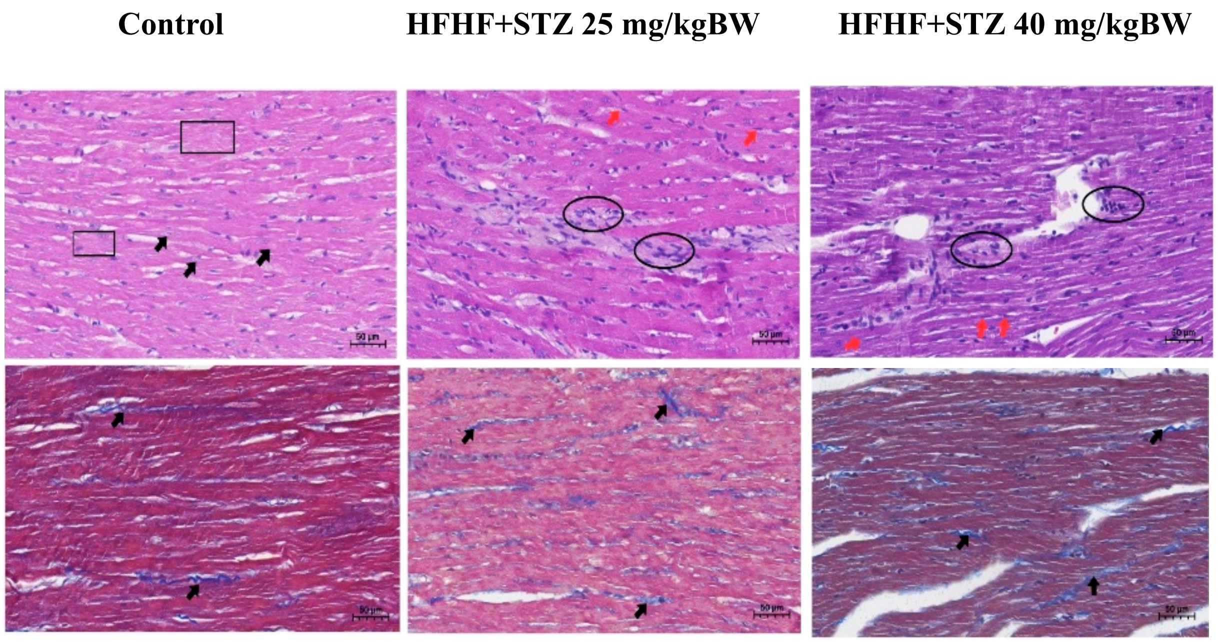

Supplementary Figure S1. Representative myocardial histopathological findings from the pilot model-characterization study of the HFHF/STZ-induced early-stage diabetic cardiomyopathy model. Representative myocardial sections stained with Hematoxylin–Eosin (HE, upper panels) and Masson's Trichrome (MT, lower panels). Representative HE-stained myocardial sections from the control group and STZ-treated groups are shown. The control group demonstrates preserved myocardial architecture with uniformly shaped cardiomyocytes, eosinophilic cytoplasm, visible cross-striations (rectangles), and centrally located oval nuclei (black arrows). In the STZ-treated groups, myocardial architecture shows progressive alterations, including irregular arrangement of cardiomyocytes, variation in cell size and shape, and inflammatory cell infiltration (ovals). Increased numbers of fibroblasts are also observed (red arrows), accompanied by reduced cytoplasmic striations and nuclear changes. (Hematoxylin–Eosin ×200). Representative Masson's Trichrome–stained myocardial sections demonstrate minimal interstitial collagen deposition in the control group, whereas the STZ-treated groups show increased blue-stained collagen within the interstitial and perivascular areas. The extent of collagen deposition appears greater in the STZ 40 mg/kg group than in the STZ 25 mg/kg group, indicating a graded pattern of fibrotic remodeling across STZ doses. (Masson's Trichrome ×200).

Supplementary Table S3. Baseline characteristics of the 8-day cohort

Parameter	DCM	DCM + 5-MTP 25 mg	DCM + 5-MTP 50 mg	DCM + 5-MTP 100 mg	P value
Body length (cm)	19,3 ± 0,9	19,9 ± 1,3	20,3 ± 2,2	19,3 ± 1,0	0,703
Body weight (g)	19,3 ± 0,9	19,9 ± 1,3	20,3 ± 2,2	19,3 ± 1,0	0,703

Fasting blood glucose (mg/dL)	99,3 ± 9,3	100,0 ± 10,7	98,0 ± 16,6	88,3 ± 10,7	0,511

Pre-STZ induction

Post-STZ induction

Parameter	DCM	DCM + 5-MTP 25 mg	DCM + 5-MTP 50 mg	DCM + 5-MTP 100 mg	P value
Body length (cm)	20,8 ± 1,4	20,9 ± 1,3	21,9 ± 0,9	20,4 ± 0,5	0,311
Body weight (g)	308,5 ± 98,0	299,5 ± 59,8	256,5 ± 45,4	266,0 ± 45,3	0,633
Fasting blood glucose (mg/dL)	249,5 ± 101,9	213,5 ± 53,0	201,8 ± 3,6	221,3 ± 93,2	0,824

Supplementary Table S4. Baseline characteristics of the 16-day cohort

Pre-STZ induction

Parameter	DCM	DCM + 5-MTP 25 mg	DCM + 5-MTP 50 mg	DCM + 5-MTP 100 mg	P value
Body length (cm)	19,8 ± 1,9	19,1 ± 0,6	19,3 ± 1,0	19,8 ± 1,0	0,832
Body weight (g)	229,5 ± 25,1	223,0 ± 17,5	219,5 ± 6,4	215,5 ± 4,4	0,648
Fasting blood glucose (mg/dL)	100,5 ± 12,0	113,0 ± 8,6	86,3 ± 26,1	89,3 ± 14,3	0,148

Post-STZ induction

Parameter	DCM	DCM + 5-MTP 25 mg	DCM + 5-MTP 50 mg	DCM + 5-MTP 100 mg	P value
Body length (cm)	21,8 (20,5 – 22,0)	20,0 (20,0 – 21,0)	20,8 (20,0 – 21,5)	20,5 (20,0 – 22,0)	0,156
Body weight (g)	232,5 ± 17,9	262,0 ± 46,7	277,0 ± 55,2	227,0 ± 22,8	0,264
Fasting blood glucose (mg/dL)	244,8 ± 141,2	213,5 ± 39,0	274,5 ± 54,7	204,0 ± 49,4	0,621

Supplementary Table S5. Baseline characteristics of the 32-day cohort

Pre-STZ induction

Parameter	DCM	DCM + 5-MTP 25 mg	DCM + 5-MTP 50 mg	DCM + 5-MTP 100 mg	P value
Body length (cm)	19,3 ± 1,5	19,9 ± 0,9	19,0 ± 0,8	19,3 ± 1,7	0,797
Body weight (g)	233,5 ± 22,1	226,5 ± 28,9	233,0 ± 6,2	214,0 ± 12,4	0,483
Fasting blood glucose (mg/dL)	94,3 ± 22,3	93,3 ± 14,8	95,5 ± 13,2	91,3 ± 11,8	0,984

Post-STZ induction

Parameter	DCM	DCM + 5-MTP 25 mg	DCM + 5-MTP 50 mg	DCM + 5-MTP 100 mg	P value
Body length (cm)	21,0 (21,0 – 22,0)	20,8 (20,0 – 22,0)	20,5 (19,0 – 21,0)	21,0 (20,0 – 22,0)	0,417
Body weight (g)	209,5 ± 38,9	266,5 ± 32,6	275,5 ± 20,5	239,5 ± 14,2	0,026*
Fasting blood glucose (mg/dL)	289,8 ± 106,1	230,3 ± 49,8	284,5 ± 91,4	209,8 ± 89,1	0,498

Supplementary Table S6. Detailed univariate analyses (Tests of Between-Subjects Effects) for individual parameters contributing to significant multivariate analysis of variance (MANOVA) models across treatment durations.

Time Point	Parameter	Partial η^2	p-value
Day 8	TGF-β	0.714	0.001
	SMAD3	0.502	0.034
	AKT nuclear	0.350	0.147
Day 16	Caspase-3	0.774	<0.001
	NF-κB cytoplasmic	0.583	0.012
	SMAD3	0.603	0.009
	AKT cytoplasmic	0.576	0.014
	MT score	0.545	0.020
